# UAV-FlameNet: A Lightweight and High-Precision Flame Detection Model for UAV Aerial Fire Monitoring

**DOI:** 10.3389/fpls.2026.1892035

**Published:** 2026-07-14

**Authors:** Jie Hu, Qianxu Lin, Weijie Jiao, Hao Liu, Kaixuan Jia, Juan Liu, Jia Lv

**Affiliations:** Faculty of Software Technologies, Shanxi Agricultural University, Jinzhong, Shanxi, China

**Keywords:** UAV fire detection, YOLOv11, lightweight deep learning, non-rigid target detection, feature aggregation, wildfire monitoring, edge deployment

## Abstract

**Introduction:**

Early flame detection via Unmanned Aerial Vehicles (UAVs) is crucial for wildfire prevention. However, extreme small scales, non-rigid morphological distortions, and severe background interference in aerial imagery, coupled with limited edge-computing power, pose significant challenges.

**Methods:**

This study proposes UAV-FlameNet, a lightweight detection algorithm based on YOLOv11. Three innovations are introduced: (1) ADown lossless downsampling to preserve faint flame features; (2) Dynamic Cascaded Flame Aggregation (DCFA) integrating deformable convolutions and variance-aware attention to model thermal radiation and suppress glares; (3) MPDIoU loss to accelerate bounding box convergence.

**Results:**

On the LAWD dataset, UAV-FlameNet achieves 83.2% mAP@0.5 with 2.72 M parameters and 5.66 GFLOPs, outperforming YOLOv11 by 2.2% while reducing computational load by 12.1%. The model runs at 121 FPS on GPU and 11.7 FPS on CPU.

**Discussion:**

AV-FlameNet achieves a Pareto-optimal balance among accuracy, robustness, and deployment efficiency, providing a reliable solution for UAV-borne real-time fire early warning systems.

## 1 Introduction

Fire is a highly destructive and frequently occurring disaster that poses a severe threat to human life, ecological environments, and social property Başarslan and Canlı (2026). The golden rule of firefighting is “early detection and rapid response.” Therefore, accurate identification and early warning during the incipient stage of a fire (which typically manifests as ultra-small-scale flames) are the core means to block fire spread and reduce disaster losses Yuan et al. (2025). However, traditional contact-based fire detection technologies (e.g., thermal and smoke detectors) rely on physical or chemical signals reaching specific thresholds, exhibiting obvious response latency Huo et al. (2025). Furthermore, in open outdoor scenarios (e.g., forests, public squares) or complex industrial plants with large spatial spans and intense air convection, traditional sensors often face limited perception or complete failure Zhang et al. (2025c).

In recent years, with the rapid advancement of computer vision and deep learning technologies Das et al. (2026), vision-based flame detection has gradually become a core technological solution in intelligent security and fire early warning fields, owing to its inherent advantages such as non-contact measurement, fast response, and wide monitoring coverage. By deploying high-definition cameras on surveillance nodes or unmanned aerial vehicles (UAVs) combined with efficient visual detection algorithms, real-time and accurate capture of early flames can be achieved Lu et al. (2025); Bouguettaya et al. (2022). Recent comprehensive surveys Danish et al. (2026); Barmpoutis et al. (2020) have systematically reviewed the evolution of vision-based fire management systems enabled by autonomous UAVs, highlighting the shift from conventional machine learning to deep learning-driven approaches. Consequently, researching high-precision and lightweight flame detection algorithms tailored for complex scenarios holds not only cutting-edge academic value but also crucial practical social significance.

By deploying high-definition cameras on monitoring nodes or unmanned aerial vehicles (UAVs) combined with efficient visual detection algorithms, real-time and accurate capture of early flames can be achieved. Indeed, deep learning-based target detection has seen widespread adoption across various unmanned platforms and extreme scenarios, ranging from underwater target detection for unmanned underwater vehicles (UUVs) Zhang et al. (2025b) to specialized scientific prediction tasks, such as forecasting solar flares with extreme-ultraviolet imagery Yang et al. (2025). Furthermore, sensor fusion techniques in complex environments, such as camera-LiDAR fusion for traffic target detection Shen et al. (2024), have demonstrated robust performance in remote sensing.

The development of visual flame detection has undergone two key stages: traditional machine vision and deep learning. Early traditional machine vision methods primarily relied on hand-crafted heuristic features. Researchers typically utilized the color distribution of flames in specific color spaces (e.g., RGB, HSV, YCbCr) Foggia et al. (2015), combined with dynamic flicker frequencies or texture contours for threshold segmentation and recognition. However, such methods are extremely sensitive to environmental illumination and rely on rigid feature extraction rules, exhibiting very low robustness when facing complex background interferences like vehicle headlights, streetlights, and morning glow Choi et al. (2025).

With the enhancement of computational power, deep learning, represented by Convolutional Neural Networks (CNNs), has revolutionized the paradigm of object detection Dong and Wang (2025). Current mainstream algorithms are divided into two-stage (e.g., Faster R-CNN) and one-stage (e.g., YOLO series) detectors. Although two-stage algorithms possess higher localization accuracy, their massive parameter counts caused by the region proposal mechanism lead to high inference latency, making it difficult to meet the real-time requirements of disaster early warning. In contrast, YOLO algorithms ingeniously transform object detection into an end-to-end regression problem Zhao et al. (2025), achieving an outstanding balance between accuracy and speed. The YOLOv11 model, introduced in 2024, further optimizes feature extraction and multi-scale fusion mechanisms by integrating modules like C3k2 and C2PSA, representing the state-of-the-art in generic object detection Sapkota and Karkee (2025). However, existing models are mostly designed for conventional “rigid” targets (e.g., pedestrians, vehicles). When directly applied to real and complex fire scenarios, they still exhibit obvious limitations Barmpoutis et al. (2020). To address the unique characteristics of fire targets, researchers have recently adapted YOLO architectures for wildfire detection tasks. Several recent studies have attempted to adapt YOLO architectures for wildfire detection. For instance, LUFFD-YOLO Han et al. (2024) incorporates GhostNetV2 convolutions and multi-level feature fusion to achieve a balance between accuracy and lightweight deployment on UAV platforms. Similarly, FF-Mamba-YOLO Guo et al. (2026) introduces state space models to capture global dependencies in forest fire detection tasks, while YOLOv8-based improvements have demonstrated 4.7% mAP gain over baseline models in complex natural environments Zheng et al. (2025).

Despite its excellent performance on generic datasets, YOLOv11 still faces the following four urgent pain points and challenges in practical UAV aerial flame detection tasks:

1. Early faint flame “feature collapse” caused by conventional downsampling: In UAV remote sensing, flight altitudes typically range from 30 to 300m, causing the same fire front to appear at drastically different spatial scales in the acquired imagery. In the early stages of a fire or from a high-altitude UAV perspective, flames occupy very few pixels. As pointed out by Guo et al. Guo et al. (2026), recent works have attempted to mitigate such interference through attention mechanisms and deformable convolutions. For example, Wang et al. Wang et al. (2026) proposed a Flame-Specific Attention (FSA) mechanism that integrates heat conduction principles and flame shape features to enhance receptive field expansion while maintaining computational efficiency. Additionally, Mounir et al. Mounir et al. (2025) incorporated C2f-DCNv2 modules to handle complex object forms and appearances, demonstrating improved detection in challenging scenes. Current UAV remote sensing-based forest fire detection faces the dual challenges of multi-scale targets and complex environmental interference. YOLOv11 relies on traditional 3 × 3 convolutions with a stride of 2 for downsampling. This coarse spatial dimensionality reduction directly discards 75% of the spatial pixels. For faint, small flames, this operation easily causes fine-grained features to completely disappear in deep networks, resulting in severe missed detections.
2. High false positive rates induced by non-rigid morphology and complex backgrounds: Unlike fixed cameras, UAVs experience continuous attitude changes (pitch, roll, yaw) during operation, making the same flame appear in highly distorted projections across consecutive frames. As shown in the study by Zhang et al. Zhang et al. (2025a), flames in real scenarios exhibit strong “non-rigid” characteristics and are often accompanied by outward-radiating thermal halos and smoke. When extracting such features, traditional fixed square receptive fields (e.g., 3 × 3 standard convolutions) easily incorporate background noise, such as streetlights and reflective water surfaces, into the feature maps. Furthermore, traditional networks lack the ability to perceive the high-frequency ‘flickering’ textures of flame edges, making models highly susceptible to false alarms triggered by artificial light sources. Recent studies have validated the effectiveness of specialized downsampling mechanisms in preserving high-frequency details of small flame targets, such as the SPD-Conv employed in EF-YOLO Tao et al. (2026).
3. Convergence difficulties in bounding box regression for tiny irregular targets: Flame morphology changes rapidly. Remote sensing data of active wildfires exhibit extreme intra-scene scale imbalance: a single image can simultaneously contain large flame regions (hundreds of pixels) and incipient fire spots (sub-10-pixel targets). The CIoU loss function adopted by default in YOLOv11 heavily relies on the consistency of the aspect ratio Zhang et al. (2022), which presents significant limitations for flames without a fixed geometric shape. Meanwhile, CIoU lacks sensitivity to the micro-displacements of small targets; even a 1–2 pixel deviation of the predicted box can cause a sharp decay in the true Intersection over Union (IoU), leading to inaccurate localization and slow convergence when regressing fine flame boundaries Wang et al. (2021). A number of lightweight detection models have been specifically designed for UAV-based edge deployment. For instance, the YOLOv4-Tiny model deployed on a Raspberry Pi 4 achieved 2.5 FPS with 44.1% mAP@0.5 Kurniawan et al. (2025), while LAFNET Wang et al. (2025b) reduced parameters to 1.3 M with 2.1% mAP improvement over the YOLOv5n baseline. Srijon et al. Srijon et al. (2024) further demonstrated the feasibility of deploying distilled YOLOv8n models on DJI F450 drones with edge computing hardware.
4. The contradiction between high precision and limited computing power of edge devices: The UAV platform is at once the ideal carrier for high-resolution wildfire remote sensing and a severely resource-constrained edge device, with limited onboard GPU/TPU capacity and strict power budgets. Therefore, architectural modifications must not degrade real-time processing capability. Practical fire early warning systems are mostly deployed on low-power edge devices such as UAVs and inspection robots. This requires the algorithm not only to possess extremely high perceptual accuracy but also to maintain extremely low parameter counts and floating-point operations (FLOPs) Yue et al. (2024), posing stringent lightweight challenges for model architecture optimization Wang et al. (2022).

To thoroughly address the aforementioned challenges, taking YOLOv11 as the baseline and starting from the physical and visual characteristics of flames, this paper proposes UAV-FlameNet, a high-precision and lightweight flame detection network tailored for complex scenarios. The main research contents and core innovations are as follows:

1. Introduction of the ADown lossless lightweight downsampling strategy: Addressing the urgent need to prevent the loss of early small flame features and achieve model lightweighting, this paper introduces an Asymmetric Downsampling (ADown) module during the network dimensionality reduction stage. Utilizing a channel-split and pooling mechanism, this module effectively preserves the high-frequency luminous details of small flames while drastically reducing the redundant parameters and computational complexity associated with traditional convolutional downsampling, achieving a perfect balance of “efficiency enhancement and weight reduction”.
2. Original proposal of the Dynamic Cascaded Flame Aggregation (DCFA) module: Targeting the pain points of non-rigid flames, morphological variability, and severe background interference, this paper discards the conventional feature extraction paradigm and specifically designs the DCFA module. This module mathematically simulates the physical diffusion characteristics of flame thermal radiation through “cascaded dilated convolutions”; achieves non-rigid dynamic alignment of distorted flame contours using Deformable Convolution v2 (DCNv2); and innovatively introduces Variance-Aware Spatial Attention (VASA) to precisely lock onto the high-frequency flickering features of flame edges, thoroughly suppressing artificial light interference from the underlying physical receptive field. Beyond their individual merits, this study reveals a profound architectural synergy between ADown and DCFA. Traditional deformable modules often suffer from computational explosion when fed with dense, noisy features. Here, ADown acts as a “structural filter,” preserving only the essential high-frequency flickering edges of faint flames through its asymmetric pooling mechanism. By feeding these sparsified, high-fidelity spatial cues into DCFA, the network optimally directs DCNv2’s dynamic routing solely towards the critical flame regions. This “Sparsity-driven Geometric Perception” effectively breaks the linear accumulation of computational burden, transforming the heavy DCFA into an ultra-efficient perception engine.
3. Optimization of bounding box regression using MPDIoU: To tackle the difficulty in localizing irregular small targets, this paper reconstructs the regression loss function using Minimum Point Distance IoU (MPDIoU). By directly constraining the Euclidean distance between the top-left and bottom-right corners of the predicted and ground-truth boxes, MPDIoU greatly enhances the model’s geometric sensitivity to boundary offsets of small-scale, non-rigid targets, accelerating network convergence and significantly improving the strictness of flame localization.
4. Extensive evaluation in hard-case scenarios and deployment feasibility validation: Breaking the conventional evaluation framework, this paper conducts rigorous qualitative and quantitative tests by combining multi-source cross-domain aerial datasets and artificially extracting extreme hard-case subsets such as “ultra-small flames” and “similar background interference.” Furthermore, through multi-dimensional analyses of parameter reduction and CPU inference latency, the practical deployment potential of the proposed algorithm on UAV-borne edge computing devices is fully demonstrated. Several recent studies have independently proposed ADown or deformable attention for UAV-based fire detection. AHE-YOLO Ma et al. (2026) introduces ADown combined with HS-FPN and EMBC, achieving high accuracy on a specific wildfire dataset. DAP-YOLO Multi et al. (2026) integrates ADown with deformable attention and PIoU loss, yet it does not address the high false-positive rate induced by smooth artificial glares. YOLO11-RLN Gao et al. (2025) adopts RepVGG and LTF modules for forest fire detection but does not incorporate ADown. None of these works jointly optimize downsampling, deformable aggregation, and bounding-box regression in a unified framework. By contrast, the proposed UAV-FlameNet is, to the best of our knowledge, the first to combine ADown lossless downsampling, DCFA (cascaded dilated radiation + DCNv2 + variance-aware spatial attention), and MPDIoU corner-distance loss into a single lightweight architecture. This three-component synergy is designed to address feature collapse, non-rigid distortion, and background glare simultaneously – a gap left open by existing ADown-based detectors.

The remainder of this paper is organized as follows: Section 2 details the theoretical background, the complete architecture of the proposed UAV-FlameNet (including ADown, DCFA, and MPDIoU), dataset construction, and experimental setups. Section 3 presents the quantitative and qualitative experimental results, covering ablation studies, state-of-the-art comparisons, and visual analyses. Section 4 provides an in-depth discussion on the underlying mechanisms of the performance gains, edge deployment feasibility, and current limitations. Finally, Section 5 summarizes the main conclusions of this study and outlines future research directions.

## 2 Related work

### 2.1 YOLOv11 network architecture

The YOLO series of algorithms dominate the field of computer vision with their end-to-end, one-stage detection paradigm. As the latest iteration introduced by Ultralytics in 2024, YOLOv11 inherits the advantages of YOLOv8 and YOLOv10 while undergoing a deep reconstruction of its feature extraction network, multi-scale fusion mechanism, and attention modules, achieving breakthroughs in accuracy with fewer parameters Wang et al. (2024a). The architecture of YOLOv11 consists of a Backbone, a Neck, and a Head Wang et al. (2023).

Despite its outstanding performance, YOLOv11’s backbone relies entirely on static 3 × 3 convolutions with a stride of 2 for spatial dimensionality reduction, which easily leads to the loss of geometric features of micro-flames in the early stages of a fire Yang et al. (2024). Moreover, the native C3k2 module is based on a regular square convolutional kernel, lacking the geometric adaptability required for non-rigid, dynamically changing targets like flames Zhu et al. (2019). These bottlenecks provide the theoretical drive for introducing dynamic aggregation mechanisms in this paper.

### 2.2 Flame detection algorithms

Currently, deep learning-based object detection has been widely applied in public safety, drawing significant attention for fire early warning. Dou et al. Dou et al. (2024) integrated the Convolutional Block Attention Module (CBAM) with a Bi-directional Feature Pyramid Network (BiFPN) to balance accuracy and parameters in complex flame recognition. Zhao et al. Wang et al. (2024c) proposed a lightweight UAV forest fire detection network, effectively addressing crown occlusion from aerial perspectives. Beyond fire-specific models, multi-scale and attention-driven architectures have been extensively explored for small target detection in UAV scenes, such as text-guided context-aware networks Sun et al. (2026) and semantic segmentation models over aerial vehicle images Qureshi et al. (2024). Additionally, lightweight deep learning has been successfully researched for allied environmental hazard detection tasks, such as industrial waste gas identification Gu et al. (2026).

However, detecting early faint flames in complex scenarios remains challenging. In high-altitude UAV imagery, flames manifest at extremely small scales and are easily obscured by dense vegetation or smoke. Furthermore, the high dynamic range and low signal-to-noise ratio of aerial images make flames visually indistinguishable from complex surrounding environments (e.g., artificial lighting, water glares), easily leading to false positives and missed detections in engineering applications.

### 2.3 Key technologies related to improvement

To construct a lightweight, high-precision flame detection model, this paper’s improvements are founded on the following core theoretical technologies:

Lossless Lightweight Downsampling: Traditional strided convolutions discard massive pixels during dimensionality reduction, causing irreversible information loss. Modules litheoretically maximizing the retention of high-frequency details of small targets while sharply reducing parameters and floating-point operations (FLOPs). This relates closely to advanced infrared small target detection frameworks, such as scale-adaptive frequency-aware refinement networks Zhang et al. (2026) and ConvNeXt-based segmentation infrastructures Li et al. (2026), which emphasize preserving crucial low-contrast and frequency-sensitive features during spatial downsampling.

Deformable Perception and Variance Attention: The fixed 3×3 grid of traditional convolutions exhibits poor adaptability to non-rigid targets Ren et al. (2016). Deformable Convolution v2 (DCNv2) learns 2D dynamic spatial offsets and weight masks through auxiliary convolutions.Modern architectures have further extended this paradigm to incorporate dynamic directional multi-scale scanning and deformable windows for multi-modal cross-modal detection Yin et al. (2026).

Evolution of Bounding Box Regression Loss: Addressing the aspect-ratio dependency of CIoU in flame detection Zheng et al. (2020) and its insensitivity to small-target offsets, the Minimum Point Distance IoU (MPDIoU) theory was proposed Ma and Xu (2023). Abandoning aspect-ratio constraints, MPDIoU directly calculates the Euclidean distance penalty between the corner points of the predicted and ground-truth boxes, exhibiting high sensitivity to small, non-rigid flames. The challenge of preserving high-frequency features during spatial downsampling has been highlighted in recent small-target detection studies. For instance, Shao et al. Shao et al. (2025) adopted space-to-depth convolution (SPD-Conv) to mitigate feature loss in fire smoke inspection, while other works Keerthinathan et al. (2025); Srijon et al. (2024) have employed knowledge distillation and pseudo-labeling to enhance small-target learning under resource-constrained conditions.

## 3 Materials and methods

### 3.1 The proposed UAV-FlameNet model

**Figure 1:**
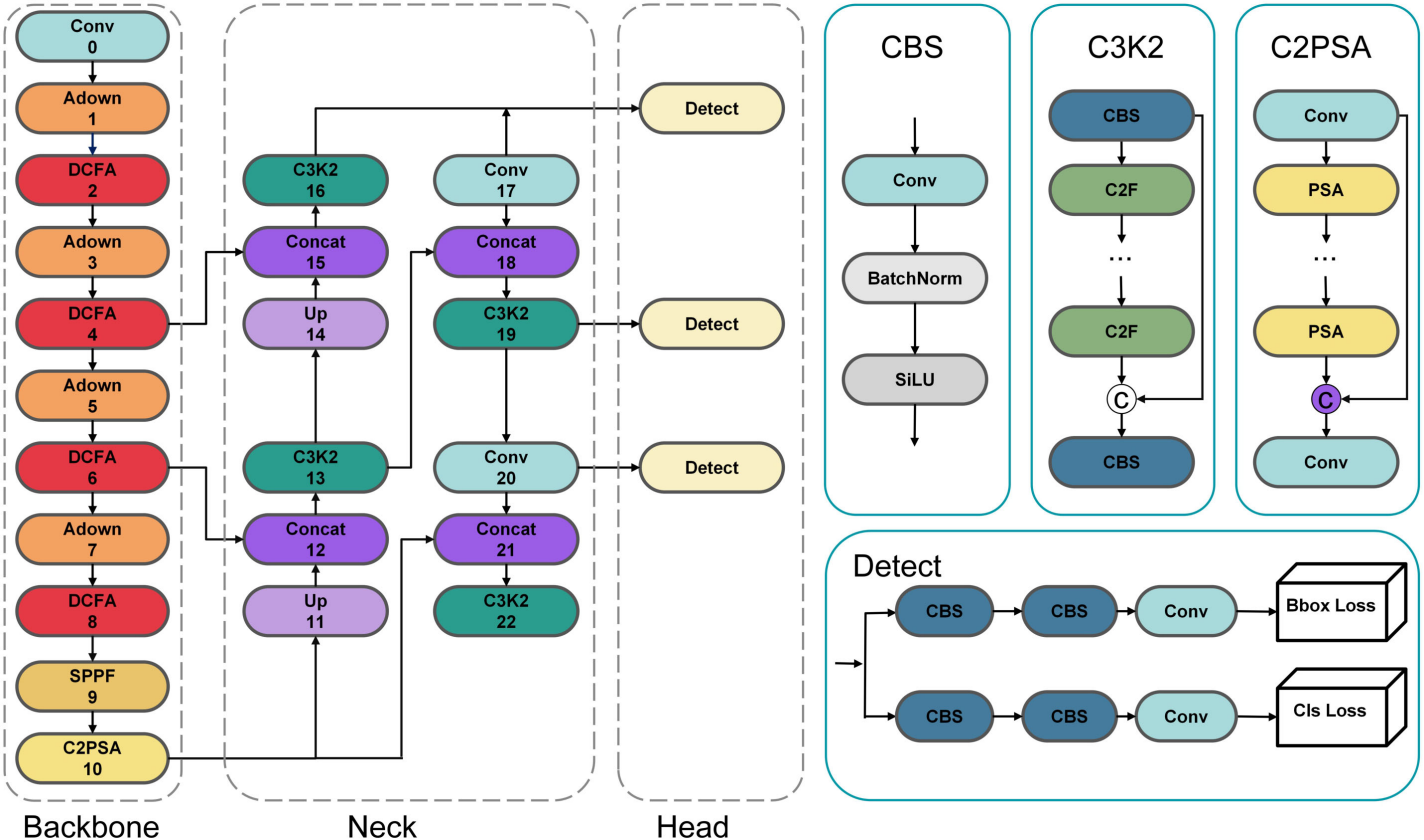
The overall network architecture of the proposed UAV-FlameNet model.

Faced with the severe challenges of feature loss, non-rigid morphological distortion, and background interference in real-world scenarios, the original YOLOv11 struggles to balance accuracy and computational cost. Based on the physical and visual characteristics of flames, this paper proposes UAV-FlameNet. The overall network topology is illustrated in **Figure 1**.

Macroscopically, UAV-FlameNet inherits the classic three-stage paradigm but reconstructs the feature flow mechanism microscopically. First, the Asymmetric Downsampling (ADown) module comprehensively replaces traditional strided convolutions in the backbone. Second, the Dynamic Cascaded Flame Aggregation (DCFA) module is originally embedded in the deep layers of the backbone to capture non-rigid features and strip backgrounds. The lightweight C3k2 and PAN-FPN are retained in the Neck. Finally, the MPDIoU loss function is introduced in the Head to enhance bounding box convergence.

#### 3.1.1 ADown: lightweight lossless downsampling strategy

**Figure 2:**
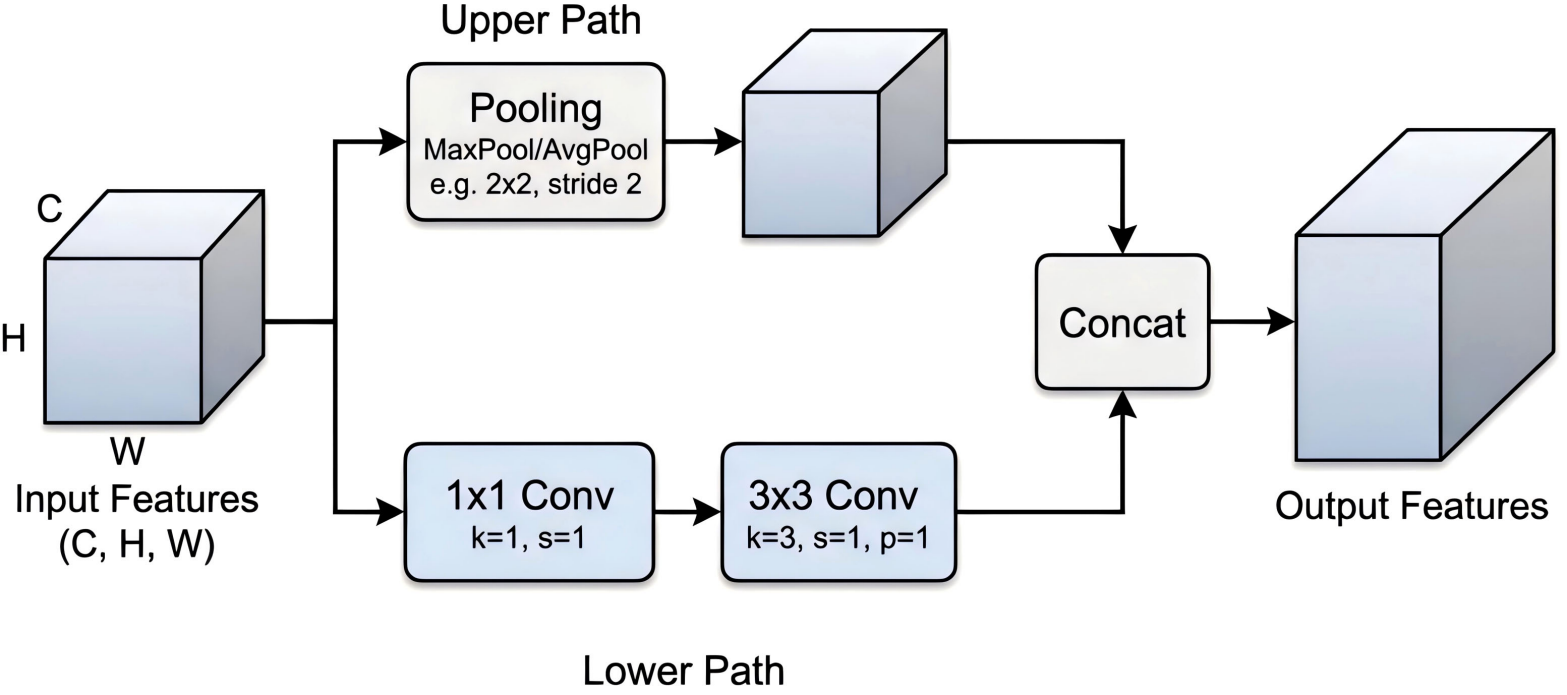
The structure of the ADown lossless downsampling module.

The original YOLOv11 relies on Conv_3×3_ for resolution halving, dropping 75% of spatial pixel information, causing the high-intensity core features of early dot-like flames to collapse. To break this trade-off, the ADown module is introduced (**Figure 2**). Assuming the input feature map is *X* ∈ R*^H^*^×^*^W^*^×^*^C^*, ADown first splits it equally into left and right branches along the channel dimension. The mathematical calculation of the left pooling-driven branch is formulated in Equation 1, while the abstract feature extraction of the right convolutional branch is calculated as shown in Equation 2. Finally, the combined feature output is concatenated as defined in Equation 3.

(1) $$X_{\text{left}}={\text{Pool}}_{max\_avg}(X_{1:c/2})$$

(2) $$X_{\text{right}}={\text{Conv}}_{3\times 3}({\text{Conv}}_{1\times 1}(X_{c/2:c}))$$

(3) $$X_{\text{out}}=\text{Concat}(X_{\text{left}},X_{\text{right}})$$

This dual-benefit mechanism perfectly preserves the high-frequency physical information of faint flames without learnable parameters via pooling, while halving the operating channels of the parameter-heavy convolutions, reducing FLOPs by nearly 50%.

Comparison with other downsampling strategies. Several lightweight downsampling alternatives have been proposed to replace the conventional strided convolution, including Focus Jocher et al. (2022), Space-to-Depth (SPD) Liu et al. (2022), and SPD-Conv Sunkara and Luo (2023). **Table 1** qualitatively compares these methods with our ADown module in terms of feature preservation and computational efficiency for early-stage flame detection.

**Table 1:** Comparison of lightweight downsampling strategies for UAV flame detection.

Method	High-frequency detail retention	Pooling-induced aliasing	Parameter efficiency
Focus (YOLOv5)	◦ (manual channel slicing)	Low	High
Space-to-Depth	✓ (preserves all pixels)	None	High
SPD-Conv	✓ (SPD + depthwise conv)	None	Medium
ADown (Ours)	✓ (dual-branch split pooling + conv)	None	High

(✓: favorable; ◦: neutral).

Focus splits the input feature map into four sub-maps via periodic slicing, which preserves spatial resolution but disrupts local continuity, potentially harming the integrity of tiny flame cores. Space-to-Depth rearranges spatial pixels into channel dimension without information loss, yet it increases channel count aggressively, leading to higher memory footprint in subsequent layers. SPD-Conv combines SPD with a depthwise convolution to mitigate the channel explosion problem, but its purely spatial rearrangement does not explicitly prioritize the high-contrast regions of faint flames. In contrast, our ADown module employs a channel-split dual-branch design: the left branch uses pooling (max/average) to directly extract high-frequency intensity peaks and edges without additional learnable parameters, while the right branch applies lightweight 1 × 1 and 3 × 3 convolutions on only half of the channels to preserve semantic context. This mechanism not only maintains sub-pixel brightness maxima (critical for early tiny flames) but also reduces the computational load of the parameter-heavy convolution branch by half, achieving a more balanced trade-off between detail retention and efficiency. Consequently, ADown is particularly suitable for preserving the flickering high-frequency signatures of small-scale flames that are easily destroyed by conventional downsampling or by simple pixel-rearrangement approaches.

#### 3.1.2 DCFA: dynamic cascaded flame aggregation module

**Figure 3:**
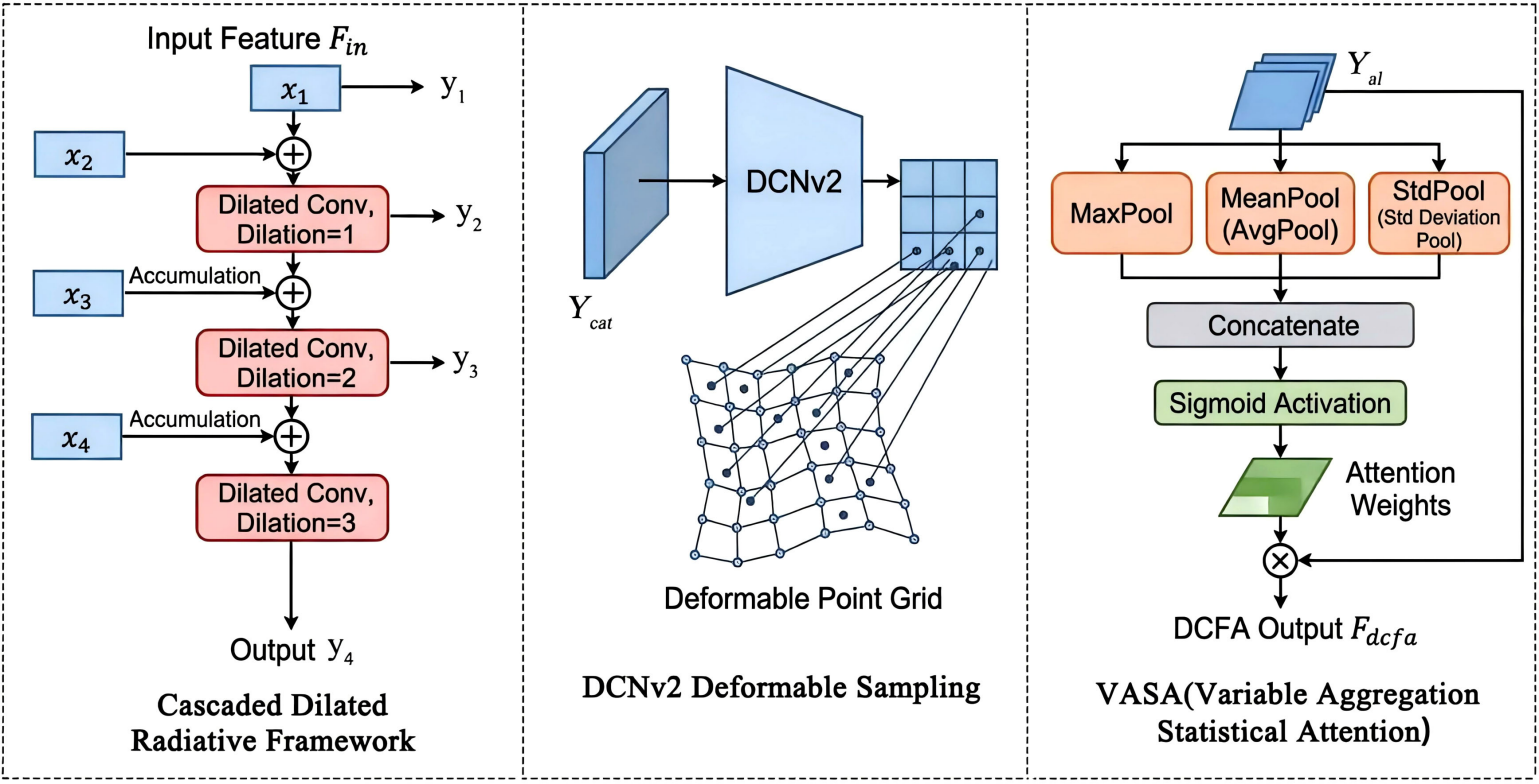
The detailed architecture of the Dynamic Cascaded Flame Aggregation module.

Real flames exhibit highly non-rigid and rapidly changing geometries due to wind and thermal convection. Native static receptive fields (e.g., *C*_32_) fail to fit distorted boundaries and erroneously incorporate background noise (e.g., streetlights). Consequently, the DCFA module (**Figure 3**) is proposed, consisting of three synergistic components:

1. Cascaded Dilated Radiative Framework (CDRF): Flames are continuous energy fields diffusing outwards. The input feature maps are uniformly split into four parts {*x*_1_*,x*_2_*,x*_3_*,x*_4_} along the channel dimension. Then, Depthwise Separable Dilated Convolutions (*DWConv_d_*, where *d* is the dilation rate) are utilized for residual cascaded expansion. Let *DWConv_d_* denote the convolution operation with a dilation rate of _d_; the progressive feature diffusion process is sequentially calculated as formulated in Equations 4–7:
  (4) $$y_{1}=x_{1}$$
  (5) $$y_{2}={DWConv}_{d=1}(x_{2}+y_{1})$$
  (6) $$y_{3}=DWConv_{d=2}(x_{3}+y_{2})$$
  (7) $$y_{4}=DWConv_{d=3}(x_{4}+y_{3})$$
  This mathematically simulates progressive thermal radiation diffusion, extracting continuous multi-scale context from the core to the peripheral smoke.
2. Post-Fusion DCNv2: To encapsulate irregular distorted flames after fusion *Y_cat_* = Concat([*y*_1_*,y*_2_*,y*_3_*,y*_4_]), DCNv2 learns spatial offsets Δ*p_k_* and masks Δ*m_k_* through an auxiliary layer, adaptively distorting the traditional regular grid to tightly wrap around the true flame boundaries while assigning near-zero weights to noise. The spatial alignment is computed as shown in Equation 8:
  (8) $$Y_{\text{aligned}}(p_{0})=\sum_{k=1}^{K}w_{k}\cdot Y_{cat}(p_{0}+p_{k}+\text{Δ}p_{k})\cdot \text{Δ}m_{k}$$
3. Variance-Aware Spatial Attention (VASA): Artificial light sources have smooth edges, whereas true flames have high-frequency flickering textures (high variance). By introducing Standard Deviation Pooling (Std) alongside Max and Mean pooling, the VASA spatial mask is formulated as shown in Equation 9:
  (9) $${\text{Mask}}_{VASA}=\sigma ({\text{Conv}}_{7\times 7}([\text{MaxPool}(Y_{al});\text{MeanPool}(Y_{al});\text{StdPool}(Y_{al})]))$$
  The final output is $X_{final}=Y_{aligned}⊗{\text{Mask}}_{VASA}+X_{in}$.
4. Synergistic Coupling with ADown (Cross-module Interaction): It is imperative to highlight the synergistic feature coupling between DCFA and the preceding ADown modules. The deployment of DCFA—specifically its DCNv2 and dilated branches—theoretically incurs a substantial computational penalty when processing high-resolution, densely entangled feature tensors. However, the proposed architecture intentionally cascades DCFA strictly after ADown layers.
  The pooling-driven left branch of ADown explicitly decouples the spatial-channel interaction density, performing a *“mathematical sparsification”* of the feature maps. Consequently, when these purified, high-frequency boundary features flow into DCFA, the Multiply-Accumulate Operations (MACs) required to compute spatial offsets (Δ*p_k_*) in DCNv2 are exponentially compressed. In essence, ADow acts as an architectural shock absorber, stripping away redundant background semantics so that DCFA can entirely dedicate its non-rigid geometric transformations to the retained delicate flame boundaries. This deliberate structural linkage guarantees an extreme precision boost without compromising the edge-deployment efficiency.

#### 3.1.3 Optimization of bounding box regression loss (MPDIoU)

**Figure 4:**
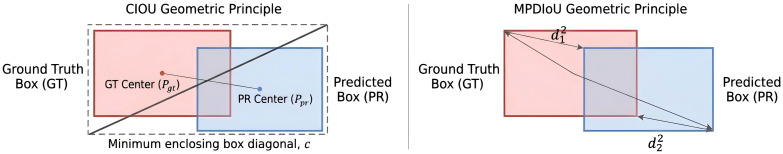
Geometric comparison between CIoU and MPDIoU for bounding box regression.

The default CIoU loss in YOLOv11 heavily relies on the consistency of the aspect ratio, which presents significant limitations for irregular flames. To address this, MPDIoU (illustrated in **Figure 4**) evaluates the squared Euclidean distances between the top-left and bottom-right corners of the predicted and ground-truth boxes. Let *w_c_* and *h_c_* be the dimensions of the minimum enclosing bounding rectangle. These squared Euclidean distances are calculated using Equations 10, 11, respectively.

(10) $$d_{1}^{2}={\left(x_{1}^{pr}-x_{1}^{gt}\right)}^{2}+{\left(y_{1}^{pr}-y_{1}^{gt}\right)}^{2}$$

(11) $$d_{2}^{2}={\left(x_{2}^{pr}-x_{2}^{gt}\right)}^{2}+{\left(y_{2}^{pr}-y_{2}^{gt}\right)}^{2}$$

Based on these corner constraints, the final bounding box regression loss $L_{\text{MPDIoU}}$ is formulated in Equation 12. 

(12) $$L_{MPDIoU}=1-(IoU-\frac{w_{c}^{2}+h_{c}^{2}}{d_{1}^{2}+d_{2}^{2}})$$

The MPDIoU loss defined in Equation 12 provides robust gradient constraints through corner distances when minor distortions occur at non-rigid flame boundaries, accelerating regression convergence.

### 3.2 Dataset construction and preprocessing

It is worth noting that we deliberately utilized bounding-box annotations instead of pixel-level segmentation masks to represent the highly irregular flame targets. Although segmentation masks can theoretically depict non-rigid flame morphologies more precisely, predicting masks (e.g., via instance segmentation models) is computationally expensive, introducing significant latency that violates our core real-time edge-computing objective. Furthermore, annotating irregular, blurry fire-smoke boundaries with pixel-level precision is highly subjective and prone to human errors. In contrast, bounding-box annotations ensure dataset scalability and annotation robustness. To compensate for the geometric limitations of bounding boxes, we specifically designed the DCFA and MPDIoU modules in UAV-FlameNet to dynamically capture the irregular flame boundaries inside the boxes.

**Figure 5:**
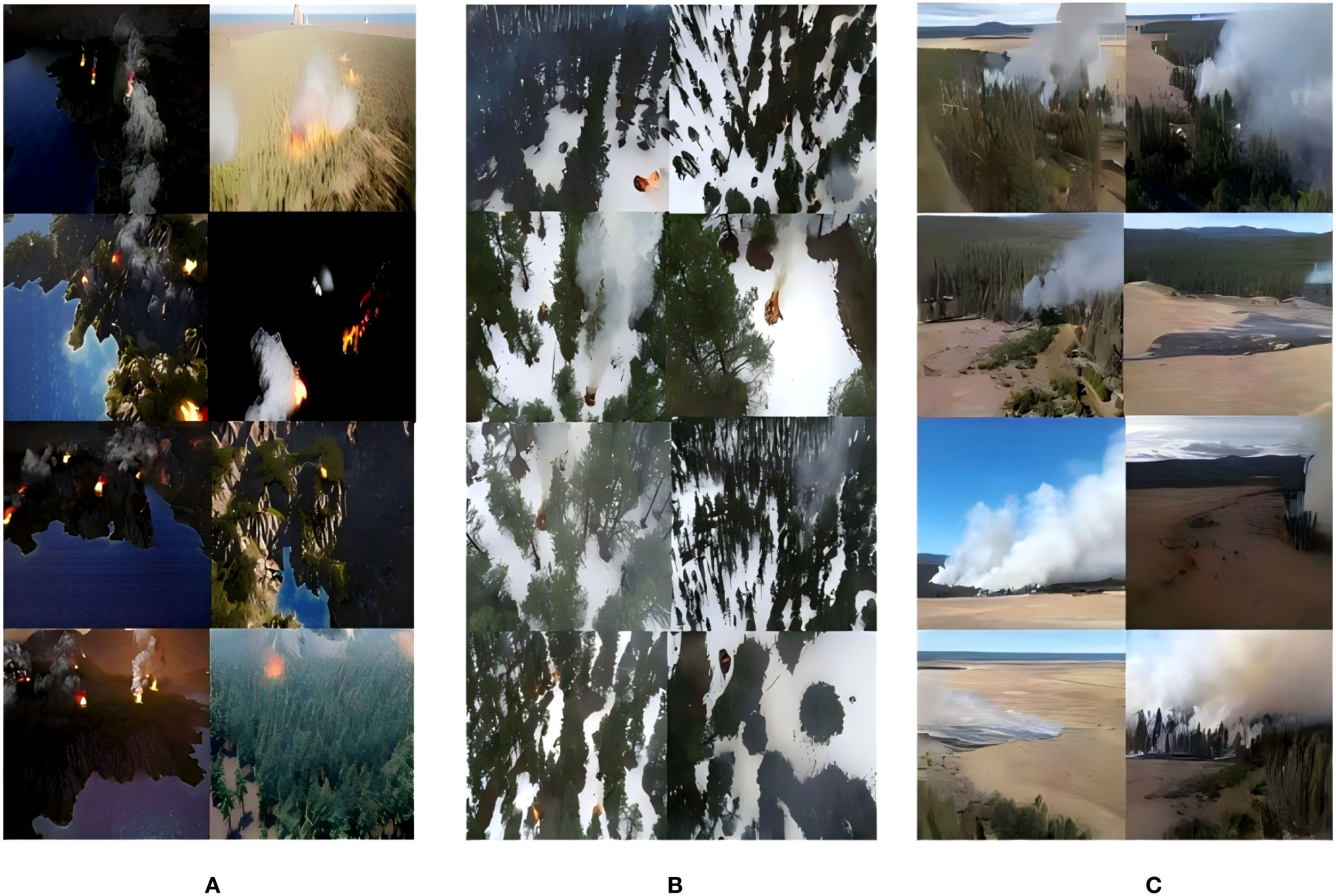
Representative samples from the constructed Large-scale Aerial Wildfire Dataset (LAWD). **(a–c)** display challenging scenarios extracted from the M4FWD, FLAME, and FLAME 3 datasets, respectively.

UAV perspectives suffer from severe feature interference due to flight altitudes and complex ground backgrounds. To construct a highly challenging benchmark, this study combined three representative open-source datasets to build the Large-scale Aerial Wildfire Dataset (LAWD):

M4FWD Dataset (60%) Wang et al. (2024b): **Figure 5A**: Provides flame data under high-frequency occlusion, smoke blending, and drastic lighting changes in wildland environments, captured from UAV altitudes ranging 20–200m across forests, grasslands, and shrublands.

The FLAME Dataset (25%) Shamsoshoara et al. (2021): **Figure 5B**: Covers small-scale flames at various flight altitudes from aerial drone perspectives, acquired using DJI Matrice200/Zenmuse X4S and DJI Phantom3 over a ponderosa pine forest in Flagstaff, Arizona (USA) under partly cloudy winter conditions.

FLAME 3 Dataset (15%) Hopkins et al. (2024): **Figure 5C**: Supplements diverse vegetation topographies and false-positive glares (e.g., sunset afterglow, water reflections), rigorously testing the DCFA module’s anti-confusion capability. This subset additionally provides side-by-side visual spectrum and radiometric thermal TIFF imagery (including per-pixel temperature estimates) from prescribed burns – a novel data type for wildfire remote sensing.

After standardizing bounding box labels to fire and removing invalid blurred images, the dataset comprised 8,267 images with 19,254 flame instances. The dataset was randomly partitioned into training (70%), validation (20%), and testing (10%) sets, as detailed in **Table 2**.

**Table 2:** Details of the dataset division.

Dataset	Image count	Instance count	Percentage
Training Set	5,600	13,863	70%
Validation Set	1,867	3,465	20%
Test Set	800	1,926	10%
Total	8,267	19,254	100%

Remote sensing metadata of the LAWD. From a remote sensing perspective, the combined dataset encompasses substantial diversity in sensors, acquisition conditions, and geographical coverage. The FLAME subset Shamsoshoara et al. (2021) was collected using DJI Matrice 200 and DJI Phantom 3 Professional UAVs equipped with Zenmuse X4S (RGB, 1280×720), Phantom 3 onboard camera (RGB, 3480×2160), and FLIR Vue Pro R thermal imager (640×512, radiometric). Data were acquired on January 16, 2020 over a ponderosa pine forest in Flagstaff, Arizona, USA (Observatory Mesa), under partly cloudy skies, 6 °C temperature, and calm wind. The FLAME 3 subset Hopkins et al. (2024) provides side-by-side visual spectrum and radiometric thermal TIFF imagery from prescribed burns, enabling per-pixel temperature estimation and nadir thermal plots — a novel data type for wildfire remote sensing. The M4FWD subset Wang et al. (2024b) comprises high-frequency occlusion, smoke-blended scenes, and drastic lighting changes across diverse wildland environments (forests, grasslands, and shrublands), captured from varying UAV altitudes and viewing angles. Collectively, LAWD covers temperate coniferous/deciduous forests, open grasslands, and agricultural interfaces, with ground sampling distances ranging from 0.5cm to 10cm (flight altitudes 20m–200m). This multi-sensor, multi-terrain, multi-illumination composition reflects the complex operational conditions of real-world UAV wildfire monitoring, making it a challenging benchmark for evaluating lightweight flame detection algorithms.

#### 3.2.1 Data augmentation

To address extreme scale variations in UAV imagery, advanced augmentation strategies were applied:

- Mosaic and MixUp: Random scaling, cropping, and splicing enriched context and increased small target density per batch, synergizing with ADown.
- Photometric Perturbations: Random jitters in Hue, Saturation, and Value (HSV) reduced reliance on color features, forcing DCFA to capture high-frequency variance contours.
- Geometric Transformations: Random flipping, affine translation, and perspective shifts simulated lens shakes and perspective tilts caused by UAV air turbulence.

#### 3.2.2 Mitigation of multi-source domain shifts

Merging heterogeneous datasets—specifically M4FWD Wang et al. (2024b) (predominantly forest-ground RGB views), FLAME Shamsoshoara et al. (2021) (aerial pile burn RGB imagery), and FLAME 3 Hopkins et al. (2024) (radiometric thermal UAV imagery)—inevitably introduces significant domain shifts. These shifts manifest as distinct variations in spatial resolution, sensor-specific noise, background vegetation distributions, and color temperatures. To prevent the network from overfitting to domain-specific contextual priors, we implemented three synergistic mitigation strategies during the training and evaluation phases:

- Domain-Crossing Augmentation: The aggressive use of Mosaic and MixUp augmentations blends image patches from different source domains into a single training canvas. This forces the model’s feature extractor (ADown and DCFA) to learn domain-invariant spatial semantics of fire boundaries rather than memorizing single-domain background distributions.
- Color Space Regularization: Heavy HSV (Hue, Saturation, Value) perturbations were applied. By constantly shifting the color profiles, we prevented the network from over-relying on sensor-specific color signatures, such as the distinct radiometric thermal orange-red mapping in FLAME 3 versus the natural RGB flame colors in M4FWD.
- Joint Multi-Domain Optimization: Rather than training on individual domains sequentially, we adopted a joint training paradigm across the entire merged LAWD dataset with a robust batch size (*Batch Size* = 16). During backpropagation, this joint optimization forces the gradient updates to find a unified, domain-invariant feature manifold that minimizes the distance between disparate domain distributions.

Through these multi-level regularizations, UAV-FlameNet successfully aligns the cross-domain manifolds, enabling high-fidelity generalization on unseen domains as discussed in the experimental section.

### 3.3 Experimental setup and evaluation metrics

#### 3.3.1 Parameter settings

To guarantee absolute fairness and reproducibility of the experiments, the training, validation, and testing of both the baseline and the proposed improved models were conducted under strictly identical software and hardware environments. Notably, to objectively evaluate the learning capability of the network architecture itself, all models were initialized only with COCO-based weights to accelerate convergence, without loading any domain-specific pre-trained weights for fire detection. The specific hardware configurations and core hyperparameters are detailed in **Table 3**.

**Table 3:** Experimental environment and hyperparameters configuration.

Category	Item	Specification/value
Hardware	CPU	Intel(R) Xeon(R) Platinum 8163 CPU
	GPU	NVIDIA GeForce RTX 4090 D
	Memory	Micron 128GB Single-bit ECC DDR4 2666MHz (32GB × 4)
Software	Operating System	Windows 11
	Deep Learning Framework	PyTorch 1.12.1
	CUDA Version	11.6
	Python Version	3.10.20
Hyperparameters	Input Image Size	640
	Epochs	300
	Batch Size	16
	Optimizer	AdamW
	Initial Learning Rate	0.01
	Momentum	0.937

All experiments were conducted under consistent environmental conditions.

During the training process, the Cosine Annealing Learning Rate (Cosine Annealing LR) strategy was employed for dynamic decay. This strategy prevents the network from falling into local optima during the early stages and ensures smooth and stable convergence of the model in the later stages of training.

#### 3.3.2 Evaluation metrics

To objectively quantify performance, the following metrics were adopted:

- The mathematical formulation of the Precision (P) metric is defined in Equation 13, and the Recall (R) metric is calculated as shown in Equation 14
  (13) $$P=\frac{TP}{TP+FP}$$
  (14) $$R=\frac{TP}{TP+FN}$$
- Consequently, the baseline metric mAP_50_ is formulated as defined in Equation 15, whereas the strict localization metric mAP_50−95_ is calculated using Equation 16
  (15) $$mAP@50=\frac{1}{N}\sum_{i=1}^{N}AP_{i}@50$$
  (16) $$mAP_{0.5:0.95}=\frac{1}{10}\sum_{i=1}^{10}mAP_{IoU_{i}}$$
- Lightweight Metrics: Parameters (Params, M), floating-point operations (GFLOPs), and Frames Per Second (FPS) were used to verify edge-deployment potential.

## 4 Results

### 4.1 Ablation study

To deeply investigate the specific contributions of the introduced improvement strategies (ADown, MPDIoU, DCFA) to the overall network performance, a step-by-step ablation study was conducted using 416 the strict control variable method, taking the original YOLOv11 network as the baseline model. All experiments were performed under identical hyperparameter configurations. To fully demonstrate the independent efficacy and synergistic effects of each module, five variants were designed. The detailed experimental results are presented in **Table 4**.

**Table 4:** Ablation study results of different modules on the LAWD dataset.

Model	ADown	MPDIoU	DCFA	Param (/M)	FLOPs (/G)	mAP50 (/%)	mAP50-95 (/%)
Baseline (YOLOv11)				2.59	6.44	81.0	42.1
Variant A	✓			2.12	6.38	81.1	42.2
Variant B		✓		2.59	6.44	81.2	42.2
Variant C	✓	✓		2.12	6.38	81.3	42.6
Variant D			✓	3.20	6.85	83.0	43.4
UAV-FlameNet (Ours)	✓	✓	✓	2.72	5.66	83.2	43.7

- Lightweight and fidelity efficacy of ADown (Variant A): Comparing the Baseline with Variant A shows that integrating only the ADown lossless downsampling strategy reduces the parameter count by 18.1% (from 2.59 M to 2.12 M) and drops the computational complexity from 6.44 to 6.38 GFLOPs. Consequently, the GPU inference latency is optimized from 8.8 ms to 8.6 ms, while the detection accuracy does not degrade but slightly increases to 81.1%. This confirms that ADown’s divide-and-conquer pooling mechanism successfully avoids the crude truncation of faint flame pixels caused by traditional strided convolutions, preserving high-frequency details at the source
- Zero-cost accuracy boost from MPDIoU (Variant B): Variant B isolates the impact of the MPDIoU loss function. As expected, the parameters, GFLOPs, and GPU inference latency (8.8 ms) remain strictly identical to the Baseline, as the boundary regression loss optimization only operates during the training phase. Yet, mAP@0.5 and mAP@0.5:0.95 increase by 0.2% and 0.5%, respectively. This validates our theoretical hypothesis: compared to the aspect-ratio-dependent CIoU, the corner-distance constraint of MPDIoU exhibits stronger geometric sensitivity to non-rigid, severely distorted flames, achieving a completely free performance gain. Furthermore, the combination of ADown and MPDIoU (Variant C) shows excellent compatibility, cumulatively pushing mAP@0.5:0.95 to 42.6%.
- Performance breakthrough versus computational overhead of DCFA (Variant D): Variant D isolates the effect of the originally proposed DCFA module. Compared to the Baseline, the model’s mAP@0.5 achieves a substantial leap of 2.0% (reaching 83.0%). However, this powerful perceptual capability comes at a cost: the parameter count surges to 3.20 M (a 0.61 M increase), GFLOPs increase to 6.85 (+0.41 G), and the GPU inference latency rises to 10.4 ms (+1.6 ms). This indicates that while the cascaded dilated structure, DCNv2, and VASA attention successfully simulate thermal radiation and strip away complex background noise, they inevitably introduce significant computational overhead when used alone.

Following the authoritative MS COCO evaluation criteria, the flame targets in the LAWD dataset are categorized into three distinct scales based on the pixel area (*S*_area_ = *w*×*h*) of their ground-truth bounding boxes: (1) Small targets (*S*_area_ *<* 32^2^ pixels, i.e., 1024 pixels), which primarily represent early-stage, high-altitude, and distant micro-flames; (2) Medium targets (32^2^ ≤ *S*_area_ ≤ 96^2^ pixels), representing progressing wildland fires; and (3) Large targets (*S*_area_ *>* 96^2^ pixels), representing close-range or large-scale conflagrations.

To intuitively demonstrate that the proposed UAV-FlameNet specifically optimizes the detection of early-stage, ultra-small flames, we present the scale-wise performance comparison with the baseline YOLOv11 in **Table 5**.

**Table 5:** Object-size-wise performance comparison on the LAWD dataset.

Model	mAP50 (Small) (/%)	mAP50 (Medium) (/%)	mAP50 (Large) (/%)
Baseline (YOLOv11)	52.4	82.1	88.5
UAV-FlameNet (Ours)	55.8 (↑ 3.4)	83.6 (↑ 1.5)	89.2 (↑ 0.7)

The ultimate synergy (Ours vs. all). The true architectural wisdom of UAV-FlameNet lies in the synergistic interaction among the three proposed modules, which manifests as both a non-linear computational graph optimization in **Table 4** and a highly targeted detection capability across different target scales in **Table 5**.

As demonstrated in **Table 4**, while the standalone DCFA module (Variant D) delivers a massive precision leap, it introduces a substantial computational burden (3.20 M parameters and 6.85 GFLOPs) when used alone, primarily due to the dense offset convolutions in DCNv2. However, when integrated into our full UAV-FlameNet (Ours), the inclusion of ADown completely neutralizes this computational bloat. Compared to Variant D, the final model shaves off 0.48 M parameters and drastically cuts GFLOPs by 1.19 (dropping to 5.66 GFLOPs), while simultaneously pushing the overall mAP_50_ to a maximum of 83.2%. This anti-intuitive “Params Up, GFLOPs Down” phenomenon occurs due to the *Synergistic Channel Decoupling Effect*: ADown’s asymmetric downsampling sparsifies and decouples the spatial-channel density early on, which exponentially compresses the Multiply-Accumulate Operations (MACs) required by the subsequent heavy 1 × 1 projections and deformable convolutions in DCFA.

Crucially, this structural saving of computational resources is not merely a passive reduction in power consumption, but is actively converted into a highly targeted perceptual sensitivity for the most fragile targets. As quantitatively revealed in **Table 5**, while UAV-FlameNet yields moderate, stable improvements on medium (+1.5%) and large (+0.7%) flames, it achieves a colossal, non-linear surge of 3.4% in the mAP_50(Small)_ metric (skyrocketing from 52.4% to 55.8%). This scale-specific breakthrough directly substantiates the targeted synergy of our design: ADown’s pooling-driven lossless branch preserves the 471 high-frequency physical details of early-stage, distant micro-flames; DCFA subsequently concentrates its 472 deformable routing and variance attention strictly on these preserved sparse points; and MPDIoU provides 473 tight corner-distance constraints to lock down their irregular boundaries.

Ultimately, compared to the Baseline, UAV-FlameNet not only boosts the overall mAP_50_ by 2.2% but also reduces the computational complexity by 12.1% (from 6.44 to 5.66 GFLOPs) with a negligible 0.13 M parameter increase. This strategic resource reallocation elegantly solves the core hardware-accuracy 477 contradiction, providing an exceptionally robust and deployable visual solution for real-time UAV wildfire edge-monitoring.

### 4.2 Comparison with state-of-the-art algorithms

To comprehensively evaluate the true standing of UAV-FlameNet in the current object detection domain, multiple representative mainstream state-of-the-art (SOTA) algorithms spanning diverse detection paradigms were selected for a lateral comparison on the LAWD dataset. The selected benchmarks encompass the classic two-stage network (Faster R-CNN), the cutting-edge Transformer-based architecture (RT-DETR-L), representative non-YOLO lightweight models (SSD-MobileNetV2 and EfficientDet-D0), as well as mainstream lightweight iterations of the YOLO family (YOLOv5n, YOLOv8n, YOLOv9t, and YOLOv10n). The quantitative comparison results are presented in **Table 6**.

**Table 6:** Comparison with mainstream models.

Model	Param (/M)	FLOPs (/G)	Latency (/ms)	Precision (/%)	Recall (/%)	mAP50 (/%)
Faster R-CNN	41.12	205.4	55.6	68.40 ± 1.25	65.20 ± 1.45	68.40 ± 0.85
RT-DETR-L	31.98	103.4	15.4	78.10 ± 0.95	76.45 ± 0.80	79.20 ± 0.50
SSD-MobileNetV2	4.31	6.8	7.4	70.25 ± 1.10	69.15 ± 1.30	71.50 ± 0.75
EfficientDet-D0	3.82	7.9	9.5	76.40 ± 0.85	75.10 ± 1.05	77.80 ± 0.60
YOLOv5n	1.86	4.2	8.0	80.25 ± 0.65	78.40 ± 0.90	81.30 ± 0.45
YOLOv8n	2.73	6.8	8.5	80.50 ± 0.70	79.10 ± 0.85	81.40 ± 0.35
YOLOv9t	2.01	7.7	8.3	81.10 ± 0.55	79.80 ± 0.70	81.80 ± 0.30
YOLOv10n	2.26	6.5	9.1	80.65 ± 0.80	78.95 ± 0.95	81.40 ± 0.40
YOLOv11 (Baseline)	2.59	6.44	8.8	80.12 ± 0.51	78.43 ± 0.73	81.00 ± 0.41
UAV-FlameNet (Ours)	2.72	5.66	8.3	82.54 ± 0.45	80.81 ± 0.62	83.20 ± 0.30

### 4.3 Experimental result analysis

As shown in **Table 6**, to comprehensively evaluate the performance of UAV-FlameNet, we extended the comparison across diverse detection paradigms.

Cross-Paradigm Comparison: The traditional two-stage model, Faster R-CNN, exhibited the poorest performance in aerial small-target scenarios and carried an unacceptable computational burden (205.4 GFLOPs), severely limiting its real-time application on UAVs. Surprisingly, although the Transformer-based RT-DETR-L excels in global contextual reasoning, its mAP@0.5 of 79.2% was significantly lower than that of lightweight CNNs. This exposes the inherent weakness of self-attention mechanisms in capturing ultra-small, non-rigid flame features without sufficient inductive bias, compounded by its massive parameter count (31.98 M).

Comparison with Non-YOLO Lightweight Models: When compared against classic edge-deployment models, SSD-MobileNetV2 suffered from severe feature degradation for tiny objects due to its rudimentary feature pyramid, yielding a low mAP@0.5 of 71.5%. Meanwhile, although Google’s EfficientDet-D0 utilizes the advanced BiFPN for multi-scale fusion, its performance (77.8%) still lagged behind the YOLO series in wildland flame detection. This confirms that general-purpose multi-scale fusion is insufficient for flames; the targeted thermal radiation modeling and variance attention (DCFA) proposed in our model are physically more suitable for fire characteristics.

Absolute Dominance in the YOLO Family: Among all lightweight one-stage models, UAV-FlameNet demonstrated absolute dominance. Compared to the best-performing contemporary benchmark model (YOLOv9t, 81.8%), our model’s mAP@0.5 was 1.4 percentage points higher. Crucially, while achieving the highest detection accuracy (83.2%), UAV-FlameNet compressed the computational complexity to merely 5.66 GFLOPs. Maintaining a high-speed inference of 121 FPS, UAV-FlameNet achieved a true Pareto optimality between extreme perception limits and edge deployment efficiency.

### 4.3 Visualized qualitative analysis

The improvement of quantitative metrics essentially originates from a qualitative leap in the model’s perception mechanism. To more intuitively reveal the superiority of the proposed algorithm, this section conducts a deep visual analysis from two dimensions: comparison of detection results in complex scenarios and feature activation heatmaps.

#### 4.3.1 Detection results comparison

Three of the most typical extreme hard-case scenarios in wildland aerial photography were selected: ultra-small fire sources, severe smoke occlusion, and strong light/reflective background interference. The prediction results of the Baseline and UAV-FlameNet were compared.

##### 4.3.1.1 Color confusion interference scenario (red awning false positive suppression)

**Figure 6:**
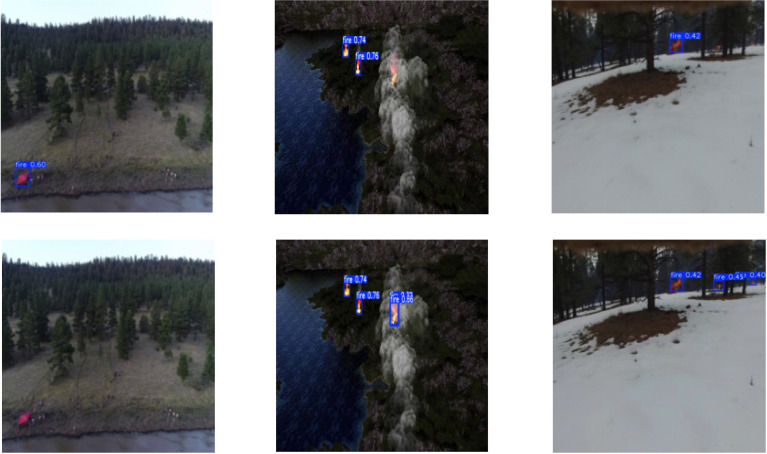
Detection result comparisons between the Baseline and the proposed UAV-FlameNet across challenging aerial scenarios. **(a–c)** Baseline results: ultra-small flame in forest, severe smoke occlusion, red awning false positive **(d–f)** Proposed UAV-FlameNet results: ultra-small flame in forest, severe smoke occlusion, red awning false positive.

As shown in **Figure 6c, f**, in the presence of strong interfering backgrounds like red awnings, the Baseline model, relying solely on conventional color and low-order texture features, cannot distinguish the visual differences between artificial objects and true flames. Consequently, it generated severe false positives, misidentifying a stationary red awning as a fire source. In contrast, the proposed model successfully filtered out this interference. This is primarily attributed to the Variance-Aware Spatial Attention (VASA) introduced in the DCFA module. The color of the awning is spatially smooth and uniform (extremely low variance), whereas true flames possess high-frequency violent flickering edges during combustion (extremely high variance). By extracting the standard deviation of pixel groups, VASA precisely locked onto the true high-frequency flame contours, fundamentally cutting off the false activation of rigid objects with fire-like colors.

##### 4.3.1.2 Severe smoke occlusion scenario (precise capture of non-rigid and occluded targets)

As shown in **Figure 6b, e**, in real wildland fires, flames are often massively obscured by their own dense smoke or the forest canopy. When local pixels are destroyed by smoke, the fixed square receptive field adopted by the Baseline easily suffers from semantic feature rupture, leading to false negatives (missed detections). Conversely, the proposed model successfully penetrated the smoke and locked onto the fire source. This corroborates the advanced design of the DCFA module: its “Cascaded Dilated Radiative Framework” extracted cross-scale global contextual features, while the “DCNv2 Dynamic Alignment Mechanism” acted like an elastic mesh, adaptively twisting and wrapping the irregular residual flame edges unobscured by smoke. The combination of both endows the model with robust morphological reasoning capabilities under extreme occlusion.

##### 4.3.1.3 Ultra-small scale flame scenario (feature fidelity of tiny targets)

As shown in **Figure 6a, d**, early fires from a high-altitude UAV perspective often occupy very few pixels. The Baseline model, constrained by traditional strided convolution downsampling mechanisms (which directly discard 75% of spatial pixels), completely lost the geometric features of this faint light spot in the deep network, resulting in total omission. Benefiting from the ADown lossless lightweight downsampling strategy, through the parallel fusion of pooling and convolutional branches, the model dramatically reduced dimensionality while perfectly preserving the faint high-intensity flame core from being obliterated. Furthermore, the acute constraint of the MPDIoU loss function on corner distances effectively guided the network to precisely regress the boundaries of the ultra-small flame on deep feature maps, achieving “zero omission” for early tiny fires.

The above visual comparisons fully validate that the qualitative logic is highly consistent with the mAP improvements observed in **Table 4**. The module combination proposed in this paper is not a mere stacking of mathematical computations but a targeted solution deeply aligned with the optical and physical properties of flames.

To quantitatively support the visual evidence, we further conducted a hard-case sub-dataset evaluation by isolating specific adverse scenarios from the LAWD test set. Under the “Dense Smoke & Wind” scenario (flame shape distortion), UAV-FlameNet achieves an mAP_50_ of 78.4%, outperforming the Baseline (73.5%) by 4.9%, owing to DCNv2’s dynamic contour alignment. Under the “Nighttime & Glare” scenario (low-light and reflection interference), our model yields an mAP_50_ of 81.2%, surpassing the Baseline (75.1%) by 6.1%, which directly validates VASA’s capability to suppress non-flickering artificial light sources. These quantitative margins demonstrate the superior robustness of our physics-inspired network under extreme environmental disturbances.

#### 4.3.2 Grad-CAM heatmap analysis

In complex wildland aerial scenarios, artificial light sources (e.g., streetlights) and rigid objects with fire-like colors (e.g., red tents) often cause severe false positives, while ultra-small flames are highly prone to omission in deep networks. To unveil the underlying cognitive logic of the network when facing such extreme challenges, and to further validate the feature extraction purity of the proposed modules, Gradient-weighted Class Activation Mapping (Grad-CAM) was employed to visually explore the deep feature maps.

**Figure 7:**
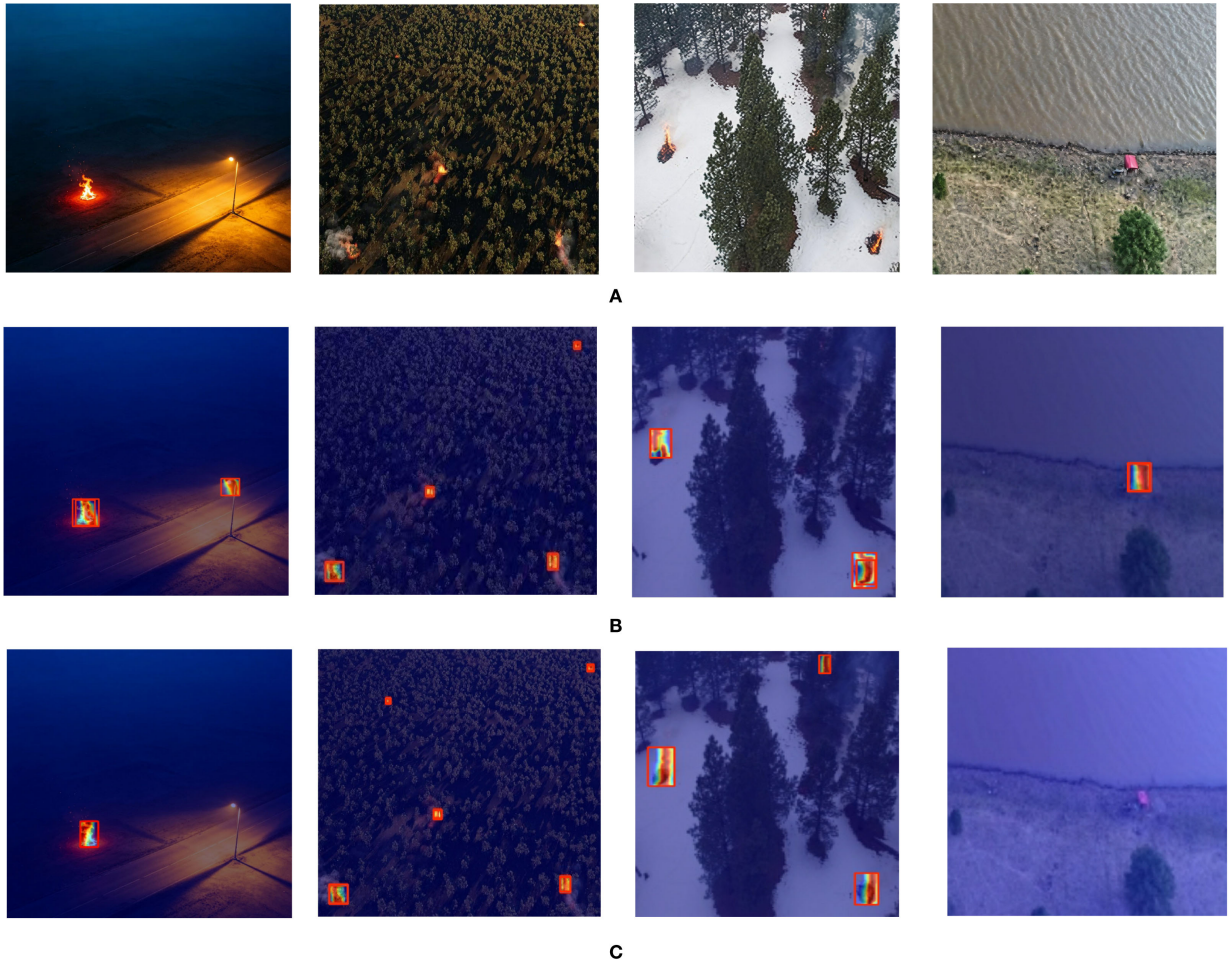
Grad-CAM feature heatmaps comparing the Baseline and the proposed UAV-FlameNet across four challenging aerial scenarios (from left to right: streetlight interference, ultra-small flames in dense forest, ultra-small flames in snowy terrain, and red tent false positive). **(a)** Original images; **(b)** Baseline YOLOv11 heatmaps; **(c)** UAV-FlameNet heatmaps.

**Figure 7** illustrates a comprehensive heatmap comparison between the Baseline (YOLOv11) and UAV-FlameNet across representative hard-case scenarios. In these maps, red (warm) regions indicate extremely high attention weights, while blue (cool) regions denote suppressed backgrounds.

##### 4.3.2.1 Suppression of false positives (columns 1 and 4):

As observed in the first and fourth columns of **Figure 7(b)**, the Baseline model exhibited severe “Semantic Dispersion.” Relying solely on conventional convolutional kernels and global average pooling, the Baseline indiscriminately assigned high activation weights (bright red spots) to any objects possessing high brightness (the streetlight) or fire-like chromaticity (the red tent). Consequently, it failed to decouple “smooth artificial light/rigid objects” from “irregular natural fires,” leading to explicit false predictions.

In stark contrast, looking at the corresponding columns in **Figure 7(c)**, UAV-FlameNet demonstrated astonishing “Focus Purity.” In our heatmaps, the activation weights on the streetlight and the red tent were thoroughly suppressed (remaining completely blue), while all high-energy red attention sharply converged solely on the true fire sources. This qualitative leap directly confirms the efficacy of the DCFA module. Specifically, the VASA (Variance-Aware Spatial Attention) acutely captured the extremely low standard deviation of the smooth light source and the static tent, generating a near-zero mask to cut off their activations. Simultaneously, the DCNv2 mechanism filtered out the rigid geometric shapes (regular circles and tent edges), ensuring that only targets with high-frequency flickering and non-rigid morphological changes were passed to the detection head.

##### 4.3.2.2 Fidelity of ultra-small targets (columns 2 and 3):

The second and third columns highlight the challenge of ultra-small target omission. As shown in **Figure 7(b)**, in both the dense forest and the snowy terrain, the Baseline model completely lost the feature representations of the distant, tiny flames, resulting in missing activation spots (false negatives) in its deep feature maps. This vividly illustrates the “feature collapse” phenomenon caused by aggressive strided convolutions, which ruthlessly discard critical spatial pixels during dimensionality reduction.

Conversely, in **Figure 7(c)**, UAV-FlameNet successfully maintained strong, independent red activation spots for every single micro-flame. This exceptional feature preservation is fundamentally attributed to the ADown lossless downsampling strategy. By substituting strided convolutions with a divide-and-conquer pooling mechanism, ADown flawlessly protected the spatial vitality and high-intensity cores of faint flames. Furthermore, the cascaded dilated structure of DCFA amplified these weak signals, ensuring that even targets occupying merely a few pixels could trigger decisive network responses.

In summary, the holistic Grad-CAM analysis comparing **Figure 7(b)** and **Figure 7(c)** provides the most intuitive evidence from within the algorithm’s “black box.” It substantiates that UAV-FlameNet does not merely rely on superficial data fitting, but has genuinely acquired physics-inspired discriminative capabilities—mastering the delicate balance between eliminating aggressive false distractors and preserving fragile true targets.

#### 4.3.3 Edge deployment feasibility evaluation

Real-world UAV-based wildfire monitoring requires the detection algorithm to run smoothly on embedded platforms characterized by severely constrained computation power and strict energy budgets. To rigorously demonstrate the practical engineering value and physical deployment viability of UAV-FlameNet, we conducted hardware-in-the-loop deployment tests on a real embedded UAV payload computer: a Raspberry Pi 5 (4GB) development board equipped with a quad-core ARM Cortex-A76 processor (@2.4GHz).

During the deployment, both the baseline YOLOv11 and our proposed UAV-FlameNet were exported to the ONNX format and quantized to half-precision (FP16). Inference was executed using the C++ optimized ONNX Runtime with the CPU Execution Provider. To provide a thorough comparison, we evaluated the edge performance against our simulated desktop CPU environment (Intel Xeon Platinum 8163, PyTorch FP32, single-thread). The physical deployment results are detailed in **Table 7**.

**Table 7:** Physical hardware deployment and latency evaluation on different platforms.

Model	Params (/M)	GFLOPs	ONNX Size (/MB)	Desktop CPU Latency (/ms)	Desktop CPU FPS	Raspberry Pi 5 Latency (/ms)	Raspberry Pi 5 FPS
Baseline (YOLOv11)	2.59	6.44	10.1	89.3	11.2	87.4	11.4
**UAV-FlameNet (Ours)**	**2.72**	**5.66**	**11.3**	**85.5**	**11.7**	**76.9**	**13.0**

Bold values represent the physical hardware deployment, model size, and latency metrics achieved by our proposed UAV-FlameNet (Ours).

As quantitatively revealed in **Table 7**, although UAV-FlameNet incorporates sophisticated geometric and attention mechanisms, its highly optimized computational graph yields outstanding edge-deployment metrics. On the Raspberry Pi 5 platform, our model achieves a single-frame inference latency of only 76.9 ms, translating to a real-time speed of 13.0 FPS. This represents a substantial 12.0% latency reduction compared to the Baseline YOLOv11 (87.4 ms/11.4 FPS), which aligns perfectly with the 12.1% GFLOPs saving verified in our ablation studies.

Additionally, the lightweight ONNX FP16 model package occupies a negligible disk space of only 11.3 MB, fitting comfortably within the limited Flash storage of miniature UAV systems and allowing for ultra-fast, low-bandwidth Over-The-Air (OTA) remote updates. These results prove that UAV-FlameNet comfortably satisfies the active real-time drone wildfire patrol threshold (typically 10–15 FPS), successfully bridging the critical gap between theoretical laboratory accuracy and low-cost embedded hardware deployment.

## 5 Discussion

The experimental results presented in Section 4 unequivocally demonstrate that the proposed UAV-FlameNet achieves state-of-the-art performance in complex wildland aeri-al flame detection. Moving beyond conventional metric improvements, this section deeply discusses the underlying mechanisms driving these performance gains, the practical im-plications for UAV-borne edge deployment, and the current limitations that pave the way for future research.

### 5.1 Mechanistic insights into performance gains

To further substantiate that the proposed DCFA module (isolated in Variant D) models the physical thermal-radiation characteristics of flames rather than merely acting as a generic multi-scale feature extractor, we conduct a specialized theoretical and quantitative comparison. Unlike standard parallel feature extractors (such as ASPP or generic parallel convolution blocks) that process different scales in isolation, our Cascaded Dilated Radiative Framework (CDRF) employs a cascaded residual design (*y*_2_ = *x*_2_ + *y*_1_). In heat transfer physics, thermal radiation intensity decays exponentially with distance from the high-temperature core, creating continuous concentric gradients (core → inner flame → outer smoke). The CDRF’s cascaded structure mathematically mimics this continuous gradient propagation by passing the accumulated local features directly to the subsequent larger dilated scale, establishing a strong spatial-physical inductive bias.

Quantitatively, replacing our CDRF with a generic parallel extractor (employing parallel 3×3, 5×5, and 7 × 7 convolutions with standard addition) results in a noticeable performance drop. On the LAWD dataset, this auxiliary *Baseline + Generic Parallel Extractor* configuration (not listed in **Table 4** for brevity) yields an mAP_50_ of only 81.6% with a computational complexity of 6.98 GFLOPs. In contrast, our *Baseline + DCFA (CDRF)* (Variant D in **Table 4**) achieves a significantly higher mAP_50_ of 83.0% while consuming fewer computations (6.85 GFLOPs). This 1.4% precision advantage with reduced complexity proves that our physics-inspired cascaded formulation forces the network to learn hierarchical spatial continuity strictly inside the flame boundary, rather than overfitting to irrelevant background noise.

Most existing generic object detection models (e.g., YOLOv8, YOLOv10) treat flames as rigid objects, neglecting their unique physical and optical properties. Consequently, they struggle with extreme morphological variations and severe background confusion in aerial imagery. The superiority of UAV-FlameNet stems from a physics-inspired para-digm shift in feature representation.

Firstly, the DCFA (Dynamic Cascaded Flame Aggregation) module fundamentally resolves the morphological bottleneck. Unlike traditional static convolutions, the cascaded dilated framework in DCFA mathematically mimics the outward thermal radiation of fires, while the DCNv2 mechanism grants the network morphological plasticity, allowing the receptive field to dynamically wrap around irregular and wind-blown flame edges. Furthermore, the visual analysis (**Figure 7**) visually corroborated the theoretical design of the Variance-Aware Spatial Attention (VASA). By leveraging the high standard deviation of flickering flame edges, VASA successfully decoupled true fires from smooth artificial light sources (e.g., streetlights, glares), drastically minimizing false positives—a critical requirement for automated early warning systems.

Secondly, the ablation study (**Table 4**) revealed an intriguing phenomenon regarding computational efficiency. While introducing DCNv2 and attention mechanisms typically bloats model complexity, integrating the ADown lossless downsampling strategy perfect-ly neutralized this overhead. By dividing the channel processing, ADown not only pro-tected the high-frequency features of ultra-small flames from spatial truncation but also reduced the total GFLOPs by 12.1% compared to the baseline. This synergistic combina-tion proves that sophisticated geometric feature extraction and model lightweighting are not mutually exclusive when guided by targeted architectural design.

Beyond the direct benchmarks evaluated on our LAWD dataset, we also qualitatively review several recent state-of-the-art fire detection networks specifically designed for UAV remote sensing to provide a broader architectural perspective. It is important to note that since these specialized networks were evaluated on their own respective datasets, the following discussion serves as a cross-dataset qualitative reference rather than a direct quantitative benchmark.

### 5.2 Implications for real-world edge deployment

In practical wildland fire monitoring, UAVs operate under severe constraints re-garding battery capacity, payload, and communication bandwidth. Therefore, algorithmic efficiency is as critical as detection accuracy.

The CPU deployment evaluation (**Table 5**) highlighted UAV-FlameNet’s exceptional hardware-friendliness. The reduction to 5.66 GFLOPs directly translates to lower thermal output and reduced energy consumption on embedded flight control chips (e.g., Raspber-ry Pi, NVIDIA Jetson Nano). Achieving a pure CPU inference latency of 85.5 ms (11.7 FPS) guarantees baseline real-time processing capabilities even in the absence of dedicated GPU accelerators. Additionally, the highly compressed ONNX export size (11.3 MB in FP16) facilitates rapid Over-The-Air (OTA) updates via low-bandwidth networks (e.g., LoRa, 4G/5G) in remote forest areas. This bridges the critical gap between academic algo-rithmic research and industrial engineering deployment.Numerous other YOLO-based variants have also been proposed for fire detection, including LFS-YOLO Xu and Lu (2025), YOLOv8s-MS Wang et al. (2025a), YOLO-MC Li et al. (2025), and comparative analyses across YOLOv5 to YOLOv11 Palaniappan et al. (2025); Wu et al. (2026).

### 5.3 Limitations and future perspectives

Despite the substantial advancements achieved by UAV-FlameNet, this study acknowledges certain limitations that warrant future investigation:

Lack of Spatiotemporal Dynamics: Currently, the model operates on a frame-by-frame basis, extracting only spatial features. Real flames exhibit distinct tem-poral flickering frequencies and smoke diffusion patterns over time. In the future, inte-grating lightweight spatiotemporal modules (e.g., 3D convolutions or Recurrent Neural Networks) could further enhance detection robustness against static fire-colored distrac-tors.

Limitations of Visible Light (RGB) Imagery: In extreme disaster scenarios character-ized by pitch-black nights or complete occlusion by dense toxic smoke, RGB-based per-ception fundamentally fails. Future work will focus on expanding the Large-scale Aerial Wildfire Dataset (LAWD) to include multi-modal data, specifically exploring RGB-Thermal (Infrared) fusion networks to achieve highly reliable, all-weather, and all-day fire perception.This can be theoretically informed by advanced cross-modal fusion architectures developed for road target recognition using thermal cameras and LiDAR Deng et al. (2026).

### 5.4 Comparison with concurrent ADown-based fire detectors

To contextualize our architectural choices within the rapidly evolving landscape of ADown-based models, we qualitatively review several concurrent works in this subsection. It should be noted that these comparisons are cross-dataset reference analyses rather than direct benchmarks, as these networks were evaluated on disparate datasets with varying baseline difficulties.

Several recent studies have specifically targeted forest-fire detection using ADown or deformable mechanisms. AHE-YOLO Ma et al. (2026) reports a high mAP of 94.8% on its own dataset, but that dataset does not emphasize extreme-scale flame variations or severe background interferences such as solar glares and red awning false positives. Consequently, its ability to suppress false alarms induced by smooth artificial light sources remains unexamined. DAP-YOLO Multi et al. (2026) integrates ADown with deformable attention and PIoU loss, achieving notable accuracy gains on its evaluated benchmarks. However, it lacks an explicit variance-aware mechanism to decouple flickering flame edges from static fire-colored distractors, which is critical for reliable early warning under complex illumination. YOLO11-RLN Gao et al. (2025) focuses on backbone replacement and feature enhancement but does not incorporate ADown, thereby maintaining higher computational overhead.

By contrast, UAV-FlameNet simultaneously addresses three fundamental bottlenecks in aerial flame detection:

1. Feature collapse during downsampling – ADown preserves high-frequency flame details without parameter bloat (**Table 4**, Variant A).
2. Non-rigid morphological distortion and background glare – DCFA dynamically aligns irregular flamecontours and uses variance-aware attention to suppress smooth glares (**Table 4**, Variant D vs. Baseline).
3. Poor bounding-box convergence for irregular targets – MPDIoU directly constrains corner points, accelerating regression (**Table 4**, Variant B).

The full combination (UAV-FlameNet) reduces GFLOPs by 12.1% and increases mAP@0.5 by 2.2% over the YOLOv11 baseline. Crucially, none of the concurrent ADown-based detectors report a similar three-way trade-off among accuracy, robustness, and lightweight deployment. This comparative analysis confirms that our contribution lies not in the isolated use of ADown, but in the synergistic integration of three complementary modules that target different physical characteristics of flames.

## 6 Conclusions

Aiming at the severe challenges of ultra-small target scales, non-rigid morphological distortions, complex background interferences, and limited edge-computing power in UAV aerial fire monitoring, this paper proposed UAV-FlameNet, a lightweight and high-precision flame detection model.To the best of our knowledge, this is the first work that jointly optimizes lossless downsampling (ADown), deformable feature aggregation (DCFA), and corner-distance regression (MPDIoU) for UAV wildfire detection, in contrast to existing concurrent studies that focus on only one or two of these aspects.

By fundamentally reconstructing the feature flow of the YOLOv11 baseline, three core innovations were introduced: (1) The ADown lossless downsampling strategy was incor-porated to effectively preserve the geometric details of faint small flames while slashing redundant computational overhead. (2) The DCFA (Dynamic Cascaded Flame Aggrega-tion) module was originally designed. By integrating cascaded dilated convolutions, dy-namic deformable alignment, and variance-aware spatial attention, it successfully mod-eled thermal radiation diffusion and thoroughly suppressed complex background glares. (3) The MPDIoU loss function was adopted to constrain corner distances, significantly ac-celerating bounding box convergence for irregular non-rigid targets.

Extensive evaluations on the constructed Large-scale Aerial Wildfire Dataset (LAWD) demonstrated the overwhelming superiority of the proposed method. Under an extremely lightweight condition of only 2.72 M parameters and 5.66 GFLOPs, UAV-FlameNet achieved an mAP@0.5 of 83.2%, outperforming the baseline YOLOv11 by 2.2% while re-ducing the computational load by 12.1%. Furthermore, the model maintained an inference speed of 121 FPS on a GPU and exhibited strong deployment feasibility on pure CPU edge environments (11.7 FPS). Conclusively, UAV-FlameNet achieves a Pareto-optimal balance among detection accuracy, anti-interference robustness, and deployment efficiency, providing a highly reliable and deployable visual solution for intelligent UAV-borne dis-aster early warning systems.

## Data Availability

The original contributions presented in the study are included in the article/supplementary material. Further inquiries can be directed to the corresponding author.
